# Identification of Prognostic Biomarkers for Bladder Cancer Based on DNA Methylation Profile

**DOI:** 10.3389/fcell.2021.817086

**Published:** 2022-01-31

**Authors:** Shumei Zhang, Jingyu Zhang, Qichao Zhang, Yingjian Liang, Youwen Du, Guohua Wang

**Affiliations:** ^1^ College of Information and Computer Engineering, Northeast Forestry University, Harbin, China; ^2^ Department of Neurology, The Fourth Affiliated Hospital of Harbin Medical University, Harbin, China; ^3^ Department of General Surgery, The First Affiliated Hospital of Harbin Medical University, Harbin, China; ^4^ Key Laboratory of Hepatosplenic Surgery, Ministry of Education, The First Affiliated Hospital of Harbin Medical University, Harbin, China; ^5^ School of Life Sciences, Anhui Medical University, Hefei, China

**Keywords:** bladder cancer, DNA methylation, protein-protein interaction network, survival analysis, prognostic markers

## Abstract

**Background:** DNA methylation is an important epigenetic modification, which plays an important role in regulating gene expression at the transcriptional level. In tumor research, it has been found that the change of DNA methylation leads to the abnormality of gene structure and function, which can provide early warning for tumorigenesis. Our study aims to explore the relationship between the occurrence and development of tumor and the level of DNA methylation. Moreover, this study will provide a set of prognostic biomarkers, which can more accurately predict the survival and health of patients after treatment.

**Methods:** Datasets of bladder cancer patients and control samples were collected from TCGA database, differential analysis was employed to obtain genes with differential DNA methylation levels between tumor samples and normal samples. Then the protein-protein interaction network was constructed, and the potential tumor markers were further obtained by extracting Hub genes from subnet. Cox proportional hazard regression model and survival analysis were used to construct the prognostic model and screen out the prognostic markers of bladder cancer, so as to provide reference for tumor prognosis monitoring and improvement of treatment plan.

**Results:** In this study, we found that DNA methylation was indeed related with the occurrence of bladder cancer. Genes with differential DNA methylation could serve as potential biomarkers for bladder cancer. Through univariate and multivariate Cox proportional hazard regression analysis, we concluded that FASLG and PRKCZ can be used as prognostic biomarkers for bladder cancer. Patients can be classified into high or low risk group by using this two-gene prognostic model. By detecting the methylation status of these genes, we can evaluate the survival of patients.

**Conclusion:** The analysis in our study indicates that the methylation status of tumor-related genes can be used as prognostic biomarkers of bladder cancer.

## Introduction

In recent years, statistical research on global diseases showed that cancer has become an important killer of human health, and seriously threatens people’s life and health. It is generally believed that cancer is caused by the accumulation of mutations in cancer susceptibility genes and resulting abnormal cell growth, but a large number of recent studies have shown that in addition to genetic variation, abnormal DNA methylation also plays an important role in the occurrence and development of cancer (Tang et al., 2018). There are many studies demonstrating the importance of DNA methylation (Mo et al., 2020; Tanaka et al., 2020; Zuo et al., 2020; Luo et al., 2021). Numerous studies have shown that global hypomethylation of DNA and hypermethylation of cytosine-phosphate-guanine (CpG)-enriched regions are common in cancers (Jaenisch and Bird, 2003; Karsli-Ceppioglu et al., 2014; Sahu and Pattanayak, 2020; Yalcin and Otu, 2021). Methylation of promoters inhibits gene transcription, and abnormal methylation is one of the main causes of genomic instability, oncogene activation and tumor suppressor gene suppression. For example, abnormal methylation in colorectal tumors is characterized by hypermethylation in promoters and transcriptional silencing of tumor suppressors or DNA repair genes (Bariol et al., 2003; Øster et al., 2011; Shen and Zou, 2020; Cheng et al., 2021), coexisting with global methylation loss that leads to chromosomal and microsatellite instability and oncogene activation (Beggs et al., 2013). Both promoter hypermethylation and global hypomethylation are markers of the early stage of colorectal cancer (Lao and Grady, 2011; Luo et al., 2014; Gao et al., 2020; Mohammed et al., 2021). Therefore, abnormal methylation may contribute greatly to the pathogenesis and progression of cancer.

Alterations in DNA methylation patterns on CpG islands were the first and most significant epigenetic abnormalities found in cancer cells. DNA methylation, an epigenetic regulator of gene expression, can lead to gene silencing. Elevated methylation of tumor suppressor genes is an early event in many tumors, suggesting that altered patterns of DNA methylation may be one of the first detectable tumor-specific changes associated with tumorigenesis. Cancer-specific methylation patterns have been found to be biomarkers for cancer. Yang et al. identified novel DNA methylation markers associated with epithelial ovarian cancer risk in a genome-wide methylation study of 63,000 women of European ancestry, and proposed that methylation of multiple CpG sites may influence epithelial ovarian cancer risk by regulating gene expression (Yang et al., 2018; Singh et al., 2021). In addition, DNA methylation has several advantages over somatic mutation analysis for cancer detection, such as higher clinical sensitivity and dynamic range. Abnormal DNA methylation can also interact with genomic and other epigenomic changes, such as histone modification and nucleosome localization during tumorigenesis and development (Feinberg et al., 2006; Esteller, 2007; Baylin and Jones, 2011). Numerous studies have demonstrated that cancer-specific methylation patterns can serve as biomarkers for cancer. Hypermethylation of the promoter region of ST18 (breast cancer suppressor gene 18) and expression loss of ST18 in tumor cells of breast cancer patients suggest that this epigenetic mechanism is a marker responsible for tumor-specific down-regulation (Jandrig et al., 2004). Currently, abnormal DNA methylation is widely studied as a disordered epigenetic mechanism in gastric cancer. For example, it has been shown that some tumor suppressor genes or tumor-related genes (such as p16, Runx3, MLH1 and CDH1) in gastric cancer can be silenced by promoter methylation (Calcagno et al., 2013). In addition, CpG island methylation also occurs in non-neoplastic gastric mucosa infected with *Helicobacter pylori* (Maekita et al., 2006; Tahara et al., 2009; Iqubal et al., 2020). Since *Helicobacter pylori* infection is closely related to the occurrence of gastric cancer (Uemura et al., 2001), the occurrence of DNA methylation in gastric mucosa after infection increases the risk of gastric cancer. All these studies confirmed the important value of DNA methylation as a tumor marker.

In addition, because abnormal DNA methylation is an early event in many tumors, the precancerous changes in these tumors were often recorded. Studies have observed a dynamic change in DNA methylation from normal gastric mucosa to precancerous lesions to gastric cancer (Lee et al., 2004). Genome-wide DNA methylation analysis for cervical intraepithelial neoplasia identified novel specific methylation markers, which can be used as a population-screening method for HPV-positive women (Boers et al., 2016). Significant epigenetic heterogeneity exists in the early precancerous liver tissues of individual patients, highlighting the potential contribution of DNA methylation to the progression of liver disease and revealing strategies for identifying epigenetic drivers of HCC (Hlady et al., 2017). An analysis of genome-wide gene expression microarray and DNA methylation microarray explored genes with abnormal DNA methylation in the early detection of breast cancer, among which HOXA4 and IGF1 were identified as increased DNA methylation in breast cancer, which may be used as biomarkers for early detection of breast cancer (Li et al., 2019; Zhuang et al., 2020; Kanathezath et al., 2021). In addition, an association between DNA methylation status and tumor survival has been demonstrated. Zhang et al. analyzed EGLNs DNA methylation data in tumor tissue samples from 1230 early-stage NSCLC patients from 5 international cohort groups and gene expression data from the Cancer Genome Atlas (TCGA) and found that EGLNs DNA methylation was associated with overall survival in early-stage NSCLC patients (Zhang et al., 2019). This suggests that DNA methylation of genes can be used as a biomarker for tumor prognosis. Therefore, it is an important research direction to interpret the occurrence and development of tumors from the perspective of epigenetic abnormalities.

Bladder cancer is a malignant tumor occurring on the bladder mucosa. It is the most common malignant tumor of the urinary system and one of the top ten common tumors. The etiology of bladder cancer is complex, including internal genetic factors and external environmental factors. A large number of studies have focused on abnormalities in DNA methylation and its important role in the occurrence and development of bladder cancer. Kawakami et al. reported for the first time that MSH3 epigenetic regulation by means of DNA methylation might contribute to gene silencing, being implicated in bladder cancer carcinogenesis (Kawakami et al., 2004). Chen et al. demonstrated that Urine DNA methylation was closely associated with early detection and recurrence monitoring for bladder cancer. They discovered 26 significant methylation markers of bladder cancer in combined analyses. They built and validated a 2-marker diagnostic model that discriminated among patients with bladder cancer with high accuracy, sensitivity and specificity (Chen et al., 2020). In addition, there are many researchers aimed of the prognosis of bladder cancer at the level of DNA methylation. Recently, with BLCA sample transcriptome data and methylation data from The Cancer Genome Atlas (TCGA), 18 target genes were identified and the signature based on them was considered an effective and independent prognostic factor (Liu et al., 2021). A meta-analysis of nineteen studies showed that patients with methylated DNA had poorer OS, compared with those with unmethylated DNA, the combined HR was 2.766 (95%CI: 2.099-3.806). Simultaneously, methylated DNA was considerably associated with shorter PFS (HR = 2.872, 95% CI: 1.971-4.185) (Yu et al., 2018). This indicated that DNA methylation was negatively correlated with the prognosis of BC patients and might become a promising biomarker. These results indicated that it is feasible and significant to analyze the prognostic markers of bladder cancer from the perspective of DNA methylation.

In this study, the DNA methylation data of bladder cancer from TCGA database were used to preliminarily obtain the potential biomarkers of bladder cancer by identifying the genes with differential DNA methylation between bladder cancer and normal control samples. However, this may not be comprehensive enough, so we further screen for potential cancer biomarkers by constructing a protein-protein interaction network. Literature excavation was conducted to confirm whether the association of these biomarkers with bladder cancer has been studied, thus proving the reliability of our screening method. Finally, we screened out prognostic biomarkers for bladder cancer by applying univariate and multivariate Cox proportional risk regression models, and divided patients into two risk groups by a prognostic index model, and verified the reliability of the prognostic model in the test set. Our study can provide a reference for clinicians to monitor patients’ survival status and timely change of treatment regimens, and provide several targets for biological experimentalists for experimental verification.

## Materials and Methods

### Data Acquisition and Processing

The Illumina Infinium HumanMethylation450 Bead Chip DNA methylation profile data of bladder cancer and clinical information and survival data of the samples were obtained from TCGA database (Cancer Genome Atlas Research Network, 2013), including 408 tumor samples, 14 normal control samples. The methylation level of each probe was represented by *β*-value, which ranges from 0 to 1, corresponding to unmethylated and fully methylated, respectively. Probes with missing data in more than 70% of the samples were removed. The remaining probes with not available (NAs) were imputed using the k-nearest neighbors (knn) imputation procedure. Unstable genomic sites, including CpGs in sex chromosomes and single nucleotide polymorphisms were removed. Because DNA methylation in promoter regions strongly influences gene expression, we selected CpGs within promotor regions. Promoter regions were defined as 2 kb upstream to 0.5 kb downstream from transcription start sites.

### Identification of Differentially Methylated Genes

DNA methylation is the most extensively documented epigenetic modification that can influence cell fate and gene expression (Jones and Baylin, 2007), which finally leads to the inhibition of gene expression through formation of heterochromatin in the gene regulatory region (Li and Zhang, 2014). In this study, identification of differentially methylated genes between cancer samples and adjacent control samples for bladder cancer is our first task.

We used *t*-test to recognize the differentially methylated CpGs between cancer patients and adjacent control tissues. Benjamini-Hochberg method was used in multiple tests to adjust the *p* values. The CpGs whose adjusted *p* value were less than 0.05 and the difference of the average of *β* were more than 10 percent were considered distinctly differentially methylated CpG between cancer patients and adjacent control tissues. All processes were programmed using R software.

### Construction of Protein-Protein Interaction Network and Extraction of Subnet

The protein-protein interaction network is composed of individual protein through the interaction between each other to participate in all aspects of the life process such as biological signal transmission, gene expression regulation, energy and material metabolism, and cell cycle regulation. This network has many important applications in bioinformatics research (Cheng et al., 2018; Liu et al., 2019; Cheng et al., 2019; Zhai et al., 2020). In this study, we chose an integrated PPI network as background, which was integrated from the Biomolecular Interaction Network Database (BIND), the Biological General Repository for Interaction Data sets (BioGRID), the Database of Interacting Proteins (DIP), the Human Protein Reference Database (HPRD), IntAct, the Molecular INTeraction database (MINT), the mammalian PPI database of the Munich Information Center on Protein Sequences (MIPS), PDZBase (a PPI database for PDZ-domains) and Reactome. Then we used the genes mapped by the DMGs (differentially methylated genes) identified above as seed gene set. They were mapped into integrated PPI network and a sub-network was extracted. The sub-network was composed of the seed genes and their one-step neighbor genes which were connecting with the seed genes in integrated PPI network. The degree of nodes in sub-network was defined as the number of genes which were connected with the specific genes. It could be used to predict the importance of identified cancer-related DMGs. Genes with larger degree are often regarded as the Hub genes in the network. The network was visualized through the Cytoscape (http://www.cytoscape.org/), an open-source software for constructing biological networks. Cytoscape is one of the most popular open-source software tools for visualizing biomedical networks composed of proteins, genes and other types of interactions. It provides a multi-functional interactive visual interface for researchers to explore complex biological interconnections supported by various annotations and experimental data, thus facilitating research tasks such as predicting gene function and constructing pathways (Su et al., 2014; Cheng et al., 2020).

### Literature Mining Confirms the Genes Identified From the Network

To test whether the biomarkers screened in our study are indeed associated with bladder cancer, we used PubMed (www.pubmed.gov) to conduct a literature search, and analyzed whether the genes were indeed related to bladder cancer in previous reports, so as to prove that the screening of tumor-related genes through the network is effective. PubMed (White, 2020) is a widely used search engine, built and maintained by the National Biotechnology Information Center (NCBI) of the National Library of Medicine (NLM), which can provide more than 28 million academic biomedical publications.

This method is simple and feasible. The selected genes and bladder cancer will be searched as the keywords in the literature database, and then consult the literature to see if there is a confirmed relationship between the screened genes and the occurrence and development of bladder cancer.

Since there are many genes obtained, one-to-one comparison is neither feasible nor necessary. Therefore, the several genes with higher degree values were selected for literature search.

### Biological Functions and Pathways Enrichment Analysis of Differentially Methylated Genes

In this study, using DAVID (Huang et al., 2009a; Huang et al., 2009b), a database used for annotation, visualization and integration of discoveries, we conducted a GO (Gene Ontology) biological functions enrichment analysis and a KEGG (Kyoto Encyclopedia of Genes and Genomes) pathways enrichment analysis towards the list of candidate biomarker genes, with *p* controlled within 0.05, which could find out the biological characteristics involved by our biomarker genes.

### The Construction of Prognostic Model and Survival Analysis

In order to be accurate, all cancer patients were divided into two data sets on average in this study, a training set and a test set. The training set was used for establishment of models and screening of prognostic markers while the test set was for follow-up validation of the prognostic model proposed by training set. The division of two sets should meet the following criteria: 1) All samples are divided into training set and test set randomly. 2) There were no significant differences in age distribution, staging, follow-up time and mortality between the two sets (Fisher’s exact test or *t* test). That is to say, patients of all types were randomly assigned to the training and test sets, including patients with missing clinical information. Then, we used the samples in training set and the candidate prognostic markers obtained from PPI network to construct models to screen for specific prognostic markers in bladder cancer.

In the first step, we found out DNA methylation spectrum of candidate markers, as well as clinical phenotype information and follow-up information of the samples and established a univariate COX proportional hazard regression model for each gene, so as to assess the association between patient’s survival and DNA methylation levels. Additionally, we also constructed univariate COX proportional hazard regression models to determine the clinical factors that significantly affect patient’s survival. In the next step, genes and the clinical factors that significantly affect survival were introduced into the multivariate COX proportional hazard regression model to find independent prognostic factors (genes). For each gene *i*, the formulas of univariate and multivariate COX proportional hazard regression models were defined as follows:

$$\text{h}{\left(t,x\right)}_{i}=h0\left(t\right)\exp\left(\beta_{methy}methy_{i}\right)$$ (1)

$$\text{h}{\left(t,x\right)}_{i}=h0\left(t\right)\exp\left(\beta_{methy}methy_{i}+\sum \beta_{clinical}clinical\right)$$ (2)

In these two formulas, $methy_{i}$ is the DNA methylation level vector of Gene *i* in all samples, *clinical* represents clinical attribute information, $\beta_{methy}$ , $\beta_{clinical}$ are the coefficients of the regression model. The positive regression coefficient indicates that the increase of methylation level is related to the increase of death risk (risk gene), while the negative regression coefficient indicates that the increase of methylation level is related to the decrease of death risk (protective gene). Univariate and multivariate COX proportional hazard regression models were constructed using function coxph() in survival R package.

After univariate and multivariate COX proportional hazard regression analysis, independent prognostic markers that were still significant were used to calculate risk scores in the training set (prognostic index model). Risk score is a linear combination of DNA methylation level and regression coefficients of these markers, representing different risk levels of patients.

The formula is as follows:

$$\text{Risk Score}=\sum_{i=1}^{\text{n}}\beta_{i}X_{i}$$ (3)

In the formula, $\beta_{i}$ is the COX regression coefficient of Gene *i* in the training set, $X_{i}$ is the methylation level of Gene *i*, *n* is the number of genes that have a significant impact on survival. Next, taking the median risk score as the threshold, the patients in the training set were divided into a high-risk group and a low-risk group. The survival difference between the two groups was analyzed, the overall survival status of patients was estimated by Kaplan-Meier method and the statistical significance of the difference was determined by log-rank test. Functions survfit() and survdiff() in survival R package were used in the process.

Then, the regression coefficients and the threshold of risk score from the training set were directly applied to the test set, and the patients in the test set were also divided into a high-risk group and a low-risk group. The prognostic difference between the two risk groups was assessed using the same method as in the training set. Besides, in order to consider the reproducibility of the prognostic biomarkers screened in this study. We searched the GEO database for an independent validation dataset GSE37816 which consisted of 18 bladder cancer patient samples and 6 normal control samples.

## Results

### Identification of Differentially Methylated Genes

**FIGURE 1:**
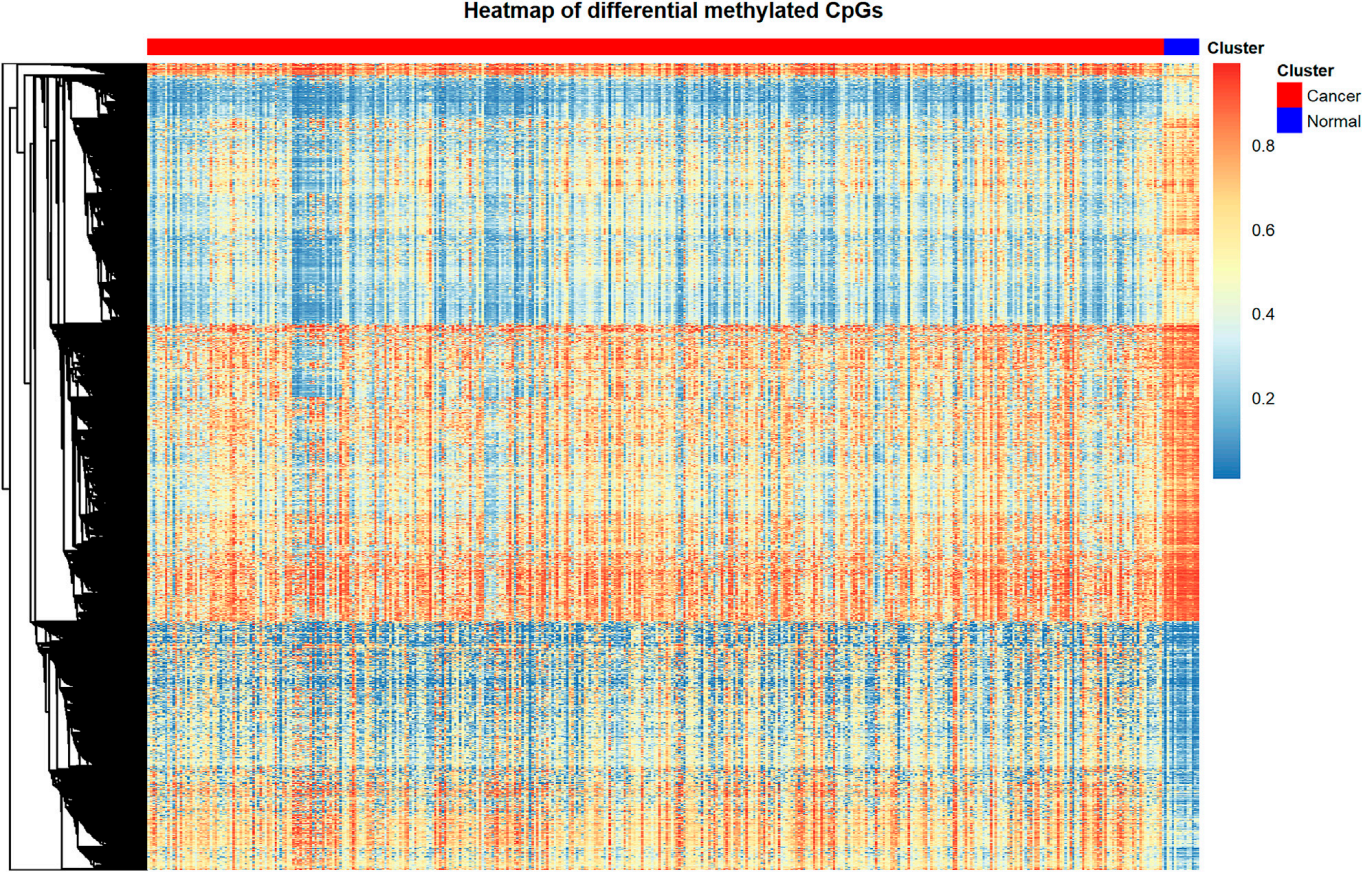
Heat map of differentially methylated CpGs.

Through the processes mentioned above, we identified 9702 differentially methylated CpGs between bladder cancer patients and control samples. The differentially methylated CpGs were shown in the heat map (Figure 1). The heat map displays the methylation level of differentially methylated CpGs in cancer samples and adjacent control samples. We utilized the function pheatmap() in pheatmap package of R to create this graph.

By using heat map to display the complex data in the form of images, it is convenient for the observers to draw fast and accurate conclusions about the internal laws of the data.

In this figure, the rows represent CpG sites, the columns represent patients, and the colors represent the levels of DNA methylation. Red represents the higher methylation level; blue represents the lower methylation level. From this figure we can see that the differentially methylated CpGs are able to separate cancer samples and adjacent control samples clearly.

### Further Potential Biomarkers of Bladder Cancer Were Obtained by Constructing Protein-Protein Interaction Network

**FIGURE 2:**
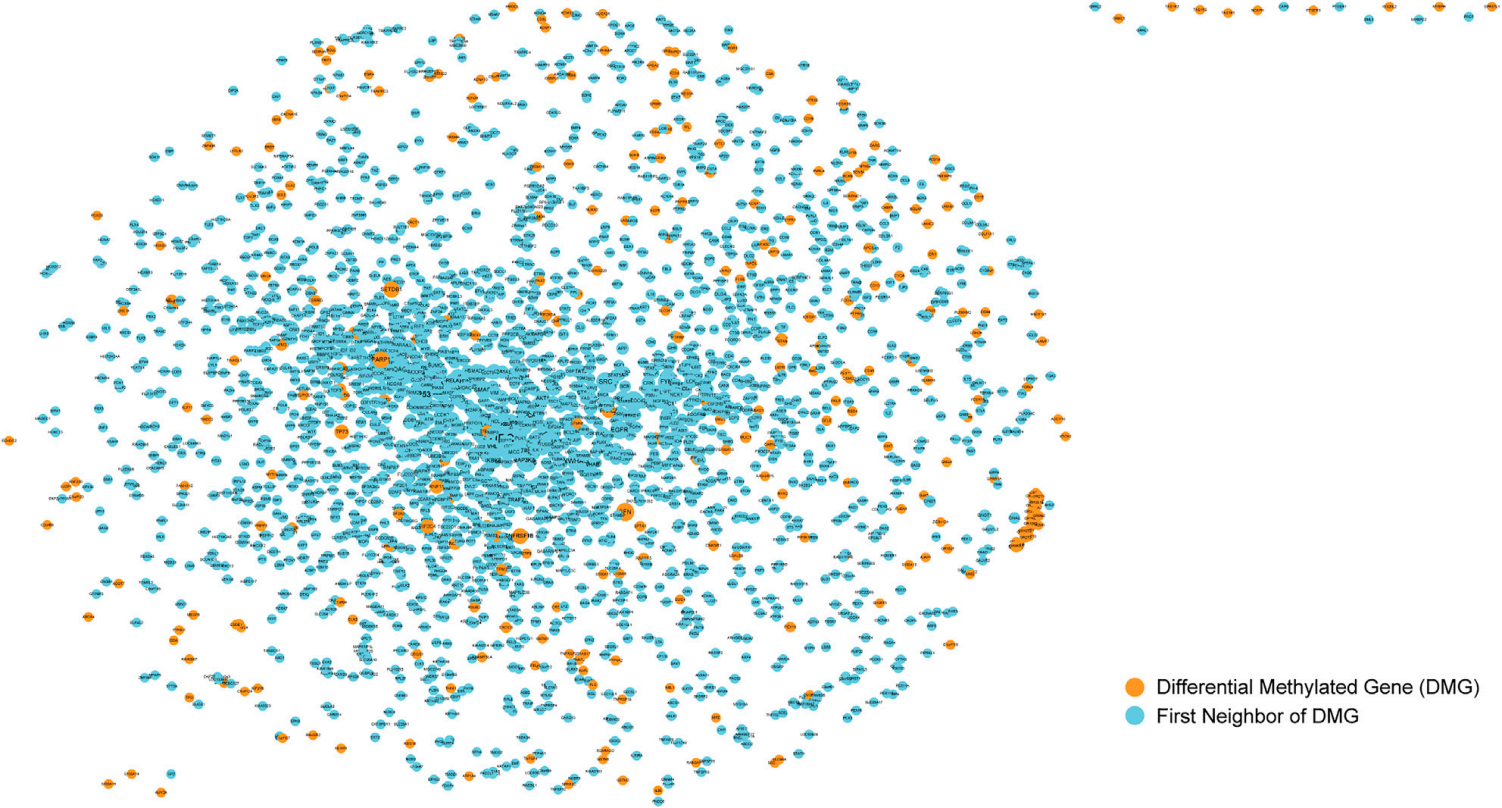
Sub-network of protein-protein interaction network. The nodes colored orange represents the differential methylated genes, and the first neighbors of them were represented by blue nodes.

The screening of cancer markers by differential methylation analysis alone may not be comprehensive and the collection of markers is huge which poses challenges for further screening of prognostic biomarkers, so we further used the physical interaction relationships of genes in the protein-protein interaction network to further screen candidate cancer markers. We used the obtained human protein-protein interaction information to construct a protein-protein interaction network (PPI). It can be seen that there were 13,368 nodes and 80,077 edges, indicating that they have interactions. Abnormalities in one gene can affect other genes linked to it, increasing the risk of diseases associated with that specific gene. Therefore, the genes linked to the differentially methylated genes we obtained previously in PPI may also have a potential impact on the occurrence and development of bladder cancer. So, after that, the differentially methylated CpGs obtained in the previous step were mapped to gene symbols and mapped into the PPI. Then we selected these nodes and their one-step neighbor genes to construct a subnet(Figure 2). It can be seen that the subnet contains 2466 nodes and 21292 edges. The size of the node is represented by its degree, the greater the degree of the node, the larger the node. The nodes colored orange represents the differential methylated genes, and the first neighbors of them were represented by blue nodes. Because the nodes with larger degree connect more nodes in the network, they are particularly important. Therefore, nodes (genes) with degree ranked in top 10% of the subnet (246 genes, Supplementary Material) were selected for further analysis to obtain prognostic biomarkers for bladder cancer.

### Bladder Cancer Related Genes Were Confirmed by Literature Review

Next, to verify that our method of screening for biomarkers for bladder cancer is reliable, we selected the top genes for literature mining and verification. To see if there is any evidence in the literature that these genes are indeed involved in the development and progression of bladder cancer. We found the following conclusions: First, TP53, a well-known tumor suppressor, is unsurprisingly associated with bladder cancer. According to a recent study, RB1 and TP53 co-mutations correlated strongly with genomic biomarkers of response to immunity checkpoint inhibitors in urothelial bladder cancer (Manzano et al., 2021). A meta-analysis provided further evidences that the expression of TP53 mutation was associated with the diagnosis efficiency of advanced bladder cancer. Higher expression of TP53 mutation was observed in the high stage of bladder cancer or the MIBC, and lower expression of TP53 mutation in the Ta stage of bladder cancer or the NMIBC. The expression level of TP53 mutation was probably a critical diagnosed biomarker in advanced bladder cancer (Liao et al., 2021).

A genome-wide study showed that YWHAZ is involved in the 8q22.3 amplicon, and its gene amplification is mainly detected in muscle invasive bladder cancer (MIBC). Immunohistochemical staining confirmed that the overexpression of YWHAZ is related to higher tumor stage, lymph node/vascular infiltration and mitotic activity. Univariate and multivariate analysis further indicated the prognostic potential of YWHAZ for more aggressive cancer types. The ectopic expression of YWHAZ in bladder cells with low endogenous YWHAZ levels enhances cell resistance to Adriamycin and cisplatin and ionizing radiation (Yu et al., 2019).

Invasive urothelial carcinoma of the bladder muscle (UCB) often recurs after radical bladder surgery. Changes in the expression of sex steroid hormone receptors are related to the prognosis of UCB tumors and may represent therapeutic goals. Erben et al. detected estrogen receptor 1 (ESR1) by quantitative reverse transcription polymerase chain reaction (RT-qPCR). They found that in patients with low AR-mRNA expression, the increase in ESR1 expression was related to the decrease in RFS and DSS. The expression status of ESR1 can further divide patients with low AR expression into subgroups with significantly reduced RFS and DSS (Erben et al., 2019).

Besides, bladder cancer is the leading cause of morbidity and mortality worldwide. Currently, immunotherapy has become a valuable treatment for bladder cancer. Tumor mutational burden (TMB) is considered to be the most commonly used biomarker for predicting immunotherapy. Zhu et al. downloaded the somatic mutation data of bladder cancer from the Cancer Genome Atlas (TCGA) and the International Cancer Genome Consortium (ICGC) data sets, and found that both cohorts contained 11 frequently mutated genes, including EP300 (E1A binding protein P300). The EP300 mutation was related to the increase of TMB, suggesting a good clinical prognosis. In addition, based on Gene Set Enrichment Analysis (GSEA) and CIBERSORT algorithm, they observed that the EP300 mutation up-regulated the signal pathway of the immune system and enhanced the anti-tumor immune response. In short, EP300 can be used as a biomarker for predicting immune response of bladder cancer (Zhu et al., 2020).

In Wang et al.’s study, compared with adjacent tissues, the P-SMAD2/3 protein was significantly increased (*p* < 0.05) in bladder cancer. the combination therapy of TGF-*β* compared with TGF, 1 + hepaCAM significantly down-regulated p-SMAD2/3, procaspase 3/8/9 and PARP, and induced bladder cancer cell apoptosis-*β* alone. The overexpression of hepaCAM prevented the translocation of p-SMAD2/3 from the cytoplasm to the nucleus in bladder cancer cells. Their findings revealed the important role of the p-SMAD2/3 pathway in hepaCAM-induced apoptosis of cancer cells, and provided valuable insights for current and future clinical trials of Ad-hepaCAM and p-SMAD2/3 (Wang et al., 2016).

The purpose of this section is to test whether the genes obtained from the previous operations are really related to the research problem selected in this topic, namely bladder cancer. Only when the results obtained were tested correctly, the next work would be meaningful. Through the literature review on these genes, it can be seen from the excavation that the genes analyzed in the above steps are meaningful and can be said to be “correct” to a certain extent. This has laid a solid and reliable foundation for the subsequent analysis of related genes. However, the relationship between the methylation levels of these genes and bladder cancer remains to be further studied, which is also the research content of this paper.

### Pathways and Biological Functions Candidate Biomarker Genes Involved

**FIGURE 3:**
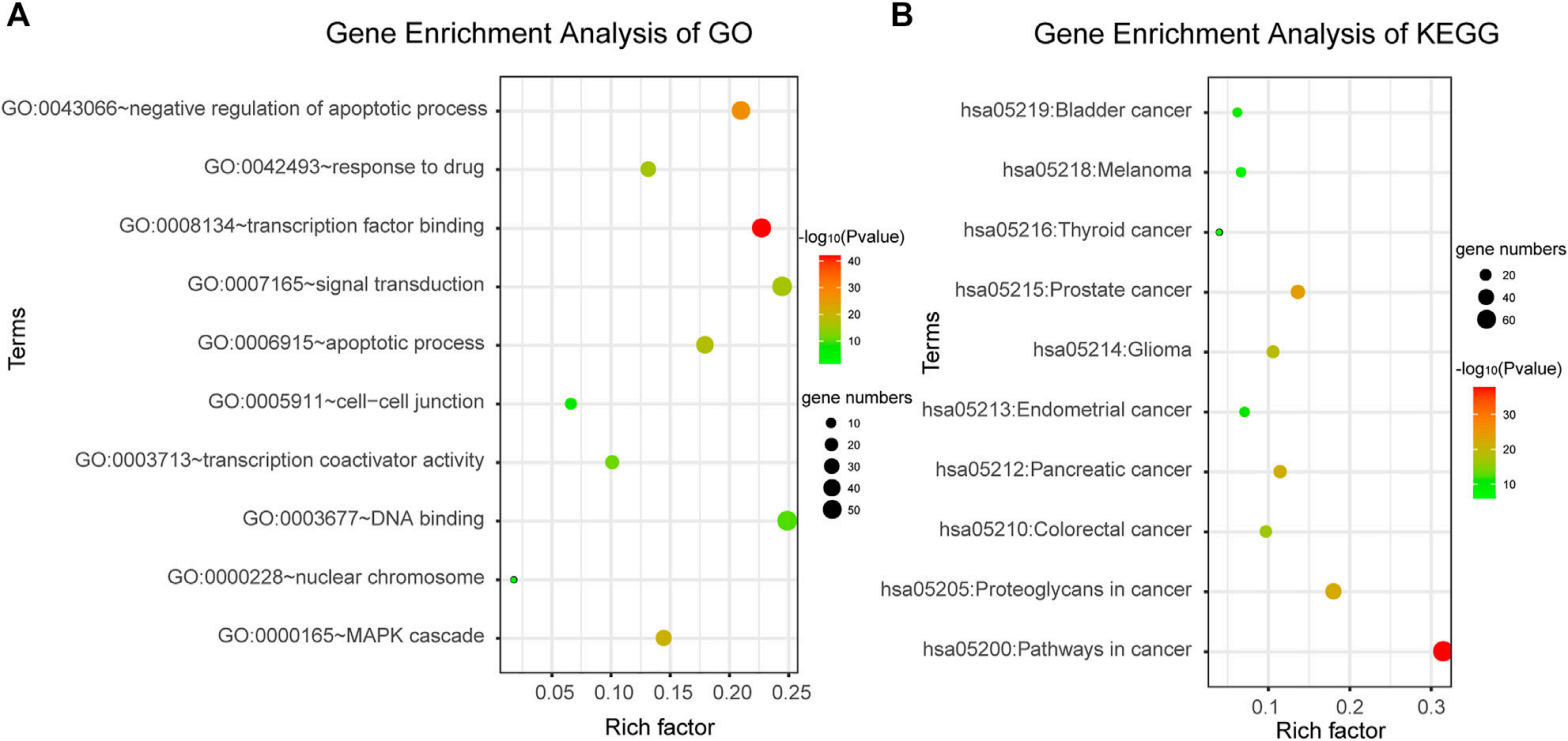
Gene enrichment analysis. **(A)** Gene enrichment analysis of Gene Ontology. **(B)** Gene enrichment analysis of KEGG.

DAVID bioinformatics tool was employed to complete the Gene Ontology and KEGG pathway enrichment analysis. From the results of enrichment, we can see that the candidate genes are involved in a number of cancer-related biological functions, such as negative regulation of apoptotic process, MAPK cascade, apoptotic process, signal transduction, response to drug, etc. (Figure 3A). And they are also involved in a number of cancer-related pathways, such as Pathways in cancer, Bladder cancer, Prostate cancer, Pancreatic cancer, etc. (Figure 3B). This suggested that the candidate genes we screened were highly likely to be cancer markers, including bladder cancer. Therefore, it was speculated that abnormalities in these genes may lead to dysregulation of related biological processes and pathways, thus inducing cancer. The results were shown in Figure 3, the color depth of each small bubble indicates the degree of enrichment. The redder the color, the higher the enrichment degree, and the greener the color, the lower the enrichment degree. Because there were too many significant terms, we only showed partial enrichment results.

### Identification and Analysis of Prognostic Markers in Bladder Cancer

Using 246 candidate biomarkers obtained from the network and according to the processes mentioned above, we identified 5 prognostic risk markers for BLCA from the univariate COX proportional hazard regression model (Table 1), including ELF3, FASLG, PRKCZ, HIST3H3, BCL10, as well as 4 clinical factors (Table 2), including age, N stage, T stage, and pathological stage. Further introduced the significant genes in the univariate analysis into the multivariate COX proportional hazard regression analysis, using the clinical factors that were significant in the above univariate analysis as covariates, 2 risk genes including FASLG and PRKCZ that independently affecting prognosis of patients were found in BLCA (Table 3).

**TABLE 1:** Univariate Cox regression results for genes.

Factor	Coefficient	*p*_value	HR	Lower95	Upper95
ELF3	1.577	0.024	4.841	1.232	19.028
FASLG	2.099	0.003	8.155	2.085	31.899
PRKCZ	−3.640	0.0002	0.026	0.004	0.189
HIST3H3	1.363	0.027	3.910	1.164	13.138
BCL10	1.784	0.001	5.950	2.024	17.493

**TABLE 2:** Univariate Cox regression results for clinical factors.

Factor	Coefficient	*p*_value	HR	Lower95	Upper95
Age	0.035	0.012	1.036	1.008	1.065
N	0.331	0.007	1.393	1.093	1.775
T	0.589	0.004	1.802	1.206	2.694
Stage	0.749	0.009	2.212	1.217	4.020

**TABLE 3:** Multivariate Cox regression results.

Factor	Coefficient	*p*_value	HR	Lower95	Upper95
FASLG	2.099	0.003	8.155	2.085	31.899
PRKCZ	−3.640	0.0003	0.026	0.004	0.189

**FIGURE 4:**
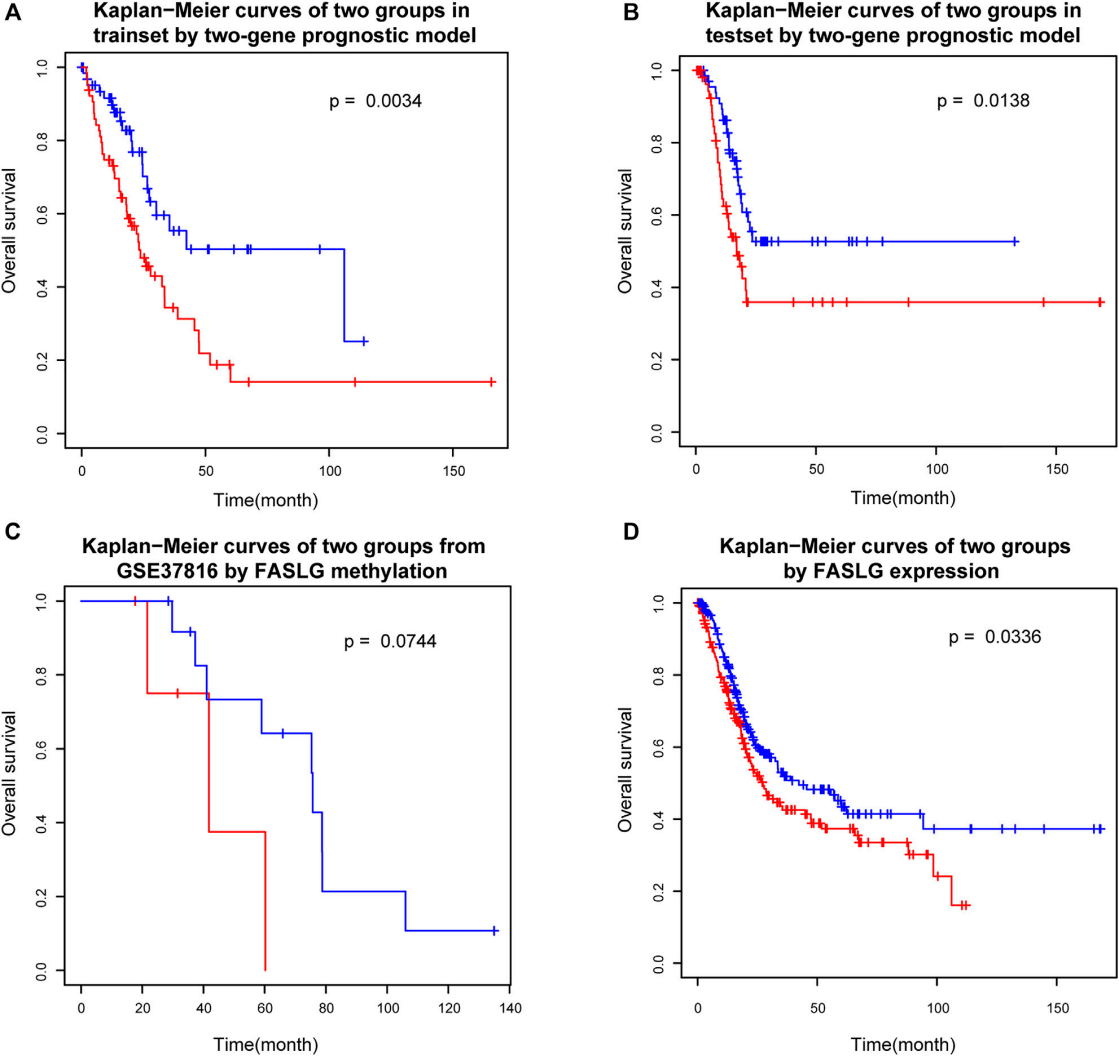
Survival analysis of two groups. **(A)** Kaplan−Meier curves of two groups in trainset by two-gene prognostic model. **(B)** Kaplan−Meier curves of two groups in testset by two-gene prognostic model. **(C)** Kaplan−Meier curves of two groups from GSE37816 by FASLG methylation. **(D)** Kaplan−Meier curves of two groups by FASLG expression.

Survival analysis of the two groups of patients which were separated by the prognostic index model (see *Materials and Methods*) showed that there were significant differences in survival between the two risk groups (Figure 4A). In order to verify the reliability of the prognostic risk markers selected in this study, the reserved test set from the previously obtained data set was used to verify the prognostic efficacy of the genes. Further validation based on the reserved test set using the method stated above showed that there were also significant differences in survival between the two groups (Figure 4B). As we can tell from the figures, the two groups of patients can be separated using the two-gene prognostic model. This suggests that the two-gene prognostic model built in this study is reliable and can be used to distinguish the high and low risk of patients.

In order to consider the reproducibility of the prognostic biomarkers identified in this study. We searched the GEO database for an independent validation dataset. However, we found only one dataset of Illumina HumanMethylation27 BeadChip data for bladder cancer, which contains patients’ survival information. The dataset accession number was GSE37816 and consisted of 18 bladder cancer patient samples and 6 normal control samples. Because there are far fewer CpG sites in Illumina HumanMethylation27 BeadChip data than in Illumina HumanMethylation450 BeadChip which was used in our study, we only found cg10161121, the CpG site mapping to one prognostic marker gene FASLG in our study, which was the only biomarker corresponding to the biomarkers in our study. Although the data also included two CpG loci mapped by PRKCZ, neither of these loci was a prognostic biomarker screened in this study. Therefore, we simply used the methylation level of cg10161121 to test its prognostic efficacy. That is, patients were divided into two groups based on the median methylation level to check if there was a difference in survival. The results showed that the difference between the survival curves of the two groups only reached borderline significance (*p* = 0.0744) (Figure 4C). To see why, we also examined the representation of this gene in the TCGA dataset (which we originally used). We only used methylation level of FASLG gene to group patients, and the results showed that the *p* value between the two groups in the training set was 0.0722, which was also only reached borderline significance, while there was no significant difference between the two groups in the test set (*p* = 0.3502), the two survival curves seem roughly distinguishable, though. This also reflects the important role of PRKCZ gene in the prognostic index model. These results suggested that single genes are not sufficient for prognostic model and that combination of these two genes are needed for a robust prognostic factor development. The combination of these two genes (to calculate the prognostic index proposed in this study) could clearly separate patients into the high and low risk groups. In any case, our study was limited by the validation data set, further validation is still necessary.

Since the DNA methylation levels of genes may affect their expression levels, we also checked the expression levels of these two genes between bladder cancer and normal samples. And we found a significant difference in PRKCZ expression between cancer and normal samples (cancer mean 9.51, normal mean 7.75, *p* value = 0.0007). However, there was no significant difference in FASLG gene expression between cancer and normal samples (cancer mean 2.96, normal mean 3.12, *p* value = 0.7017), but we can see that its expression level in the normal samples is slightly higher than in the cancer patients.

In addition, we also checked if expression of these genes can be used as prognostic biomarkers. We used the median expression levels of these two genes to divide patients into two groups and test whether there was a difference in survival between the two groups. The results showed that FASLG could divide patients into two different risk groups (*p* = 0.0366) (Figure 4D), but PRKCZ could not (*p* = 0.9099). In fact, the occurrence and progression of cancer is not always caused by abnormal gene expression, but our results further demonstrate the reliability of the FASLG gene as a prognostic biomarker gene for bladder cancer.

After consulting the literature, FASLG (Fas ligand) has been verified to be relevant with prognosis of BLCA (Mizutani et al., 2002; Ben Bahria-Sediki et al., 2018). Which also confirmed that our screening method is effective. However, the prognostic value of DNA methylation levels in this gene has not been investigated. Perhaps the methylation of this gene is intrinsically related to other omics features that need further investigation. Another gene PRKCZ has not been tested to be efficient in treating this cancer, which may be potential targets for scientists and doctors to further research on it. In addition, we also checked whether DNA methylation of these two genes has been studied in other cancers or diseases, and whether it has prognostic value in other diseases. We found that the methylation of FASLG was studied in Acute Coronary Syndrome (Li et al., 2017) and gastric cancer (Moro et al., 2020). PRKCZ DNA methylation was identified as a potential epigenetic-sensitive target in acute coronary syndrome *via* network analysis (Infante et al., 2021). It was also suggested that the PRKCZ gene was the hypermethylated gene of type 2 diabetes mellitus (T2DM) and the hypermethylation PRKCZ gene may be involved in the pathogenesis of T2DM (Zou et al., 2013). But so far, no studies have been reported on the correlation between DNA methylation of these two genes and disease prognosis.

## Discussion

Cancer is a disease with high mortality rate and a serious threat to people’s health. Previous studies focused only on the effect of genetic sequence changes on cancer, or malignancy. Recently, a relationship between cancer and the level of DNA methylation has been found. This paper studied the relationship between DNA methylation and cancer, and identified the prognostic biomarkers of bladder cancer from the perspective of DNA methylation, which can help doctors make more accurate prediction of patients’ physical conditions after treatment, which is of great value. The results, however, do not seem to be encouraging, as they may only help predict patient survival. The hope is for prevention or treatment, and perhaps future studies will find a stronger link between DNA methylation and cancer.

In this article, the data used to complete the series of operations is reliable, which is very necessary, it is the basis of the work. In this study, we not only set up a training set, but also set up a test set and an independent validation set, which were used to check whether the selected biomarkers were true and effective. This method is more convincing and also ensures the scientific nature of the research. The graphical display of the results enables researchers to have a clearer understanding of the rules and changes of the data, which can help researchers find the information contained in the data more quickly.

Based on bladder cancer as the research object, this paper analyzed the spectrum based on DNA methylation of tumor prognosis biomarkers. Through a series of analysis, the valuable conclusion has been obtained. A two-gene prognostic model (the sum of regression coefficients and methylation levels) has been proposed, and their combination allows for high and low risk grouping of patients. Using only one of them could not achieve this grouping effect, even though each of them was prognostic. But it is a relative limitation, this experiment studied only one kind of cancer, whether the conclusion is suitable for other different kinds of tumors is unknown. When the study of tumor types enough, we would discover common patterns, and perhaps conclude that cancer in general was true, and that would be valuable. It is believed that if the relevant research work can be carried on in depth, people may discover the deeper link between cancer and genes, and may be able to look at and study cancer from a new angle, so as to achieve the ultimate goal of curing cancer. Of course, the process is always hard and long, but that’s the beauty of science.

At the same time, in the process of research on this topic, the authors cannot help but think of other uses of DNA methylation. Considering its close relationship with tumorigenesis, perhaps the treatment of cancer is also involved in it. The research on this aspect is full of hope.

## Data Availability

The original contributions presented in the study are included in the article/Supplementary Material, further inquiries can be directed to the corresponding authors.

## Supplementary Material

Supplementary Table S1 Genes with degree ranked in top 10% of the subnet.
